# An open toolkit for tracking open science partnership implementation and impact

**DOI:** 10.12688/gatesopenres.12958.2

**Published:** 2019-12-04

**Authors:** E. Richard Gold, Sarah E. Ali-Khan, Liz Allen, Lluis Ballell, Manoel Barral-Netto, David Carr, Damien Chalaud, Simon Chaplin, Matthew S. Clancy, Patricia Clarke, Robert Cook-Deegan, A. P. Dinsmore, Megan Doerr, Lisa Federer, Steven A. Hill, Neil Jacobs, Antoine Jean, Osmat Azzam Jefferson, Chonnettia Jones, Linda J. Kahl, Thomas M. Kariuki, Sophie N. Kassel, Robert Kiley, Elizabeth Robboy Kittrie, Bianca Kramer, Wen Hwa Lee, Emily MacDonald, Lara M. Mangravite, Elizabeth Marincola, Daniel Mietchen, Jennifer C. Molloy, Mark Namchuk, Brian A. Nosek, Sébastien Paquet, Claude Pirmez, Annabel Seyller, Malcolm Skingle, S. Nicole Spadotto, Sophie Staniszewska, Mike Thelwall

**Affiliations:** 1 Centre for Intellectual Property and Policy (CIPP), Faculty of Law, McGill University, Montreal, QC, H3A 1W9, Canada; 2 Department of Human Genetics, Faculty of Medicine, McGill University, Montreal, QC, H3A 0C7, Canada; 3 Tanenbaum Open Science Institute (TOSI), Montreal Neurological Institute and Hospital, Montreal, QC, H3A 2B4, Canada; 4 F1000, London, W1T 4LB, UK; 5 Diseases of the Developing World, Global Health R&D, GlaxoSmithKline, Madrid, Spain; 6 Fundação Oswaldo Cruz - Fiocruz, Rio de Janeiro, RJ, 21040-900, Brazil; 7 Wellcome Trust, London, NW1 2BE, UK; 8 Montreal Neurological Institute and Hospital, Montreal, QC, H3A 2B4, Canada; 9 US Department of Agriculture Economic Research Service, Washington, DC, 20024, USA; 10 Health Research Board, Dublin, D02 H638, Ireland; 11 Arizona State University, Washington, DC, 20006, USA; 12 Sage Bionetworks, Seattle, WA, 98121, USA; 13 US National Library of Medicine, Bethesda, MD, 20894, USA; 14 Research England, UK Research and Innovation, Bristol, BS34 8SR, UK; 15 Jisc, Bristol, BS2 0JA, UK; 16 Queensland University of Technology, Brisbane, QLD, 4000, Australia; 17 The Lens, Canberra, ACT, 2601, Australia; 18 African Academy of Sciences, Karen, Nairobi, 00502, Kenya; 19 Utrecht University Library, Utrecht, CX, 3584, The Netherlands; 20 Structural Genomics Consortium (SGC), University of Oxford, Oxford, OX3 7DQ, UK; 21 Data Science Institute, University of Virginia, Charlottesville, VA, 22904, USA; 22 University of Cambridge, Cambridge, CB2 3EA, UK; 23 Alkermes, Waltham, MA, 02451, USA; 24 Department of Psychology, University of Virginia, Charlottesville, VA, 22904-4400, USA; 25 Center for Open Science, Charlottesville, VA, 22903-5083, USA; 26 Element AI, Montreal, QC, H2W 2R2, Canada; 27 GlaxoSmithKline, Stevenage, Herts, SG1 2NY, UK; 28 Warwick Research in Nursing, University of Warwick Medical School, Coventry, CV4 7AL, UK; 29 University of Wolverhampton, Wolverhampton, WV1 1LY, UK

**Keywords:** Open science, innovation, intellectual property, toolkit, performance, indicator, policy, partnership, implementation, impact

## Abstract

Serious concerns about the way research is organized collectively are increasingly being raised. They include the escalating costs of research and lower research productivity, low public trust in researchers to report the truth, lack of diversity, poor community engagement, ethical concerns over research practices, and irreproducibility. Open science (OS) collaborations comprise of a subset of open practices including open access publication, open data sharing and the absence of restrictive intellectual property rights with which institutions, firms, governments and communities are experimenting in order to overcome these concerns. We gathered two groups of international representatives from a large variety of stakeholders to construct a toolkit to guide and facilitate data collection about OS and non-OS collaborations. Ultimately, the toolkit will be used to assess and study the impact of OS collaborations on research and innovation. The toolkit contains the following four elements: 1) an annual report form of quantitative data to be completed by OS partnership administrators; 2) a series of semi-structured interview guides of stakeholders; 3) a survey form of participants in OS collaborations; and 4) a set of other quantitative measures best collected by other organizations, such as research foundations and governmental or intergovernmental agencies. We opened our toolkit to community comment and input. We present the resulting toolkit for use by government and philanthropic grantors, institutions, researchers and community organizations with the aim of measuring the implementation and impact of OS partnership across these organizations. We invite these and other stakeholders to not only measure, but to share the resulting data so that social scientists and policy makers can analyse the data across projects.

## Introduction

For the most part, people live in the safest, healthiest, richest and most democratic period in history ( Roser, 2018) partly due to the ability to secure clean water, deliver vaccines, institute the rule of law, and develop ideas of equality and democracy. Despite this, there are rising concerns about the way research is collectively organized, ranging from its escalating cost and lower research productivity ( DiMasi *et al*., 2016; Munos, 2009; Pammolli *et al*., 2011), to low public trust in researchers to report the truth even if against the interests of sponsors ( American Academy of Arts & Sciences, 2017), a lack of diversity of the players involved in the research enterprise and poor community engagement ( Puritty *et al*., 2017; Valantine & Collins, 2015), and a research culture that, among other things, provides researchers with incentives to publish over producing quality research, leading to questionable research practices and irreproducibility ( Begley & Ellis, 2012; Nosek *et al*., 2012; Open Science Collaboration, 2015). Researchers, public research organizations, firms, governments, funders and society more broadly are adopting or supporting open science (OS) practices and OS partnerships to address these concerns ( Ali-Khan *et al*., 2018b; Dai *et al*., 2018).

OS comprises a set of practices, including open education, open research funding, open access publications, open data and materials, open software tools, open infrastructures (such as digital laboratories), preregistration, and the avoidance of restrictive intellectual property. Informed by principles and values these practices aim to reduce transaction costs, promote data re-use, increase rigor and reproducibility, decrease redundant research, better involve patients, consumers and others, facilitate researcher transparency in sharing processes and results, and improve connections with a larger variety of actors to produce more innovative approaches and solutions over the medium to long terms ( Gold, 2016; McKiernan *et al*., 2016). Nevertheless, there exists no single standard for OS with the result that different organizations, governments, and firms apply OS as a label for their own favored set of practices.

This article contributes to the OS discussion by proposing the creation of an open toolkit and data set, based on internationally developed and open measures, to provide an evidence base through which we can collectively determine if, how, when, and where partnerships based on OS principles and practices can contribute to social and economic welfare in general and research and innovation (R&I) in particular. We derived the toolkit based principally on our knowledge of the life sciences but with input from other fields such as information technology and artificial intelligence. Already, the Structural Genomics Consortium (SGC) and the Montreal Neurological Institute (MNI) have agreed to use the toolkit to collect and share data. Acknowledging the different definitions of OS, we set out to measure participation in particular practices rather than determine which set of practices constitute OS.

## OS Partnerships

While there are different ways of implementing OS, we focus on partnerships (OS partnerships) in which all partners agree to comply with OS practices in conducting their joint work. Public entities, either with other public institutions or jointly with private firms, can create these partnerships by using and combining the policies, contracts, and infrastructure of institutions to increase knowledge flow and reduce redundancy ( Fecher & Friesike, 2014). Relevant public institutional policies include conditions for tenure and promotion, research grant practices, sharing by default, preregistration of studies and analysis plans, the avoidance of intellectual property rights that prevents, patient consent, continuing education and training, publication and data release ( Australian National Data Service, 2017). Contracts relate to standardized forms for material transfer, sponsorship, partnership agreements and subject participation. Institutional infrastructure comprises personnel and the physical and electronic infrastructure that support the immediate, free and usable sharing of data, software, policies, and practices ( Gold, 2016).

Through these policies, contracts, and infrastructure, those pursuing OS partnerships aim to increase efficiency and reproducibility, and inspire discovery and innovation ( Ali-Khan *et al*., 2017). Two Canadian institutions are prominent exemplars of OS private-public biomedical partnerships: the SGC and the MNI ( Dolgin, 2014; Edwards *et al*., 2009; Poupon *et al*., 2017). These build on years of open source, open access, and open data partnerships in projects such as Linux, the Apache HTTP Server Project, the Human Genome Project, the SNP Consortium ( Thorisson & Stein, 2003), and the Open Source Malaria Project, all of which have delivered significant advance in technology and knowledge.

Despite these successful partnerships, many public research organizations, government policy-makers, researchers, and firms remain uncertain about the costs and benefits of OS and their distribution among stakeholders ( Dai *et al*., 2018). The lack of evidence concerning costs and benefits as well as attitudes and experience, hinders experimentation with OS partnerships upon which to build theory around OS and R&I systems ( Ali-Khan *et al*., 2018b).

To overcome this lack of evidence, we propose here the use of a measurement toolkit to spur understanding of OS partnerships, their effects and characteristics. The toolkit consists of measures through which to collect data to be reported annually, interview guides for semi-structured interviews, sample surveys to assess implementation of OS practices, and other measures that can be collected by or for OS and non-OS partnerships. These shared quantitative and qualitative data are based on a common coding framework (See the Measurement Toolkit below). The policies comprise communication, patient and public involvement and engagement, intellectual property management, promotion and peer review criteria, skill development and training, sharing, and commercialization models. We propose that the toolkit and resource become adopted as a community-managed and open toolkit around the globe.

A critical contribution of this article is to propose that prospective data on OS partnerships be collected and shared. A prospective approach will strengthen the quality of the data and move us beyond the more common retrospectively created data sets that inevitably leave theoretical holes, rely on surrogate measures, lack historical context, and result in incomplete data sets ( Kemp & Prasad, 2017; Schwartz & Sichelman, 2017). The measurement toolkit will enable prospective collection and sharing of data on OS partnerships. As such, this measurement toolkit will provide richer, more in-depth and harmonized data to better study OS partnerships. With greater knowledge of how these partnerships contribute to R&I, we envision that policy-makers and researchers will devise better indicators of success for particular projects or funding programs.

The measurement toolkit was created with quantitative measures and qualitative approaches that research organizations participating in OS and non-OS partnerships could implement for collecting data about their collaborations. Here, we describe how we created these measures through a collaborative process drawing on the expertise of various stakeholders, including researchers, publishers, and funders. We begin with a literature review outlining the rationale for our methodology and our conceptual approach. We then describe the development of the measures. We end with a call to the larger community to comment upon and improve the proposed measures and to begin implementing them.

## Literature review

Previous studies have focused more on the practice and implementation of OS and less on the measurable effects that OS may have on better engagement, research efficiency, communications, and priority setting, as well as new delivery mechanisms and new products and services ( Jones *et al*., 2014; National Academies of Sciences & Medicine, 2018; Tripp & Grueber, 2011). For example, some initiatives present both quantitative and qualitative indicators to track openness and transparency in publication and data sharing ( Smith, 2017; Smith *et al*., 2016) and stakeholder understanding and engagement with OS ( Ali-Khan *et al*., 2017; Tuomi, 2016). Other studies developed indicators to investigate how organizations implement OS ( Lampert *et al*., 2017; Nosek *et al*., 2015; Smith, 2017; Smith *et al*., 2016; Tuomi, 2016) and a few studies have evaluated the implementation or impact of specific OS policies or practices ( Hardwicke & Ioannidis, 2018; Kidwell *et al*., 2016). Our project differs from the other studies by developing more comprehensive measures of both social and economic influence, research outcomes, diversity and inclusion, trust, and opportunities for youth and early career researchers. Our measures aim to facilitate researchers’ understanding of the nature and extent of the impact of OS. 

In addition to earlier studies on OS, other studies have proposed measures of innovation in general, such as the OECD’s Oslo Manual ( OECD/Eurostat, 2005). These measures, however, do not evaluate the relationship between OS partnerships and outcomes. Further, many of these measures are *ad hoc* to the specific studies and created based on retrospectively created data sets, limiting their use in more generic contexts. Finally, these measures tend to focus on firms using proprietary models, such as open innovation and closed/semi-closed partnerships ( Community Innovation Surveys ( Mairesse & Mohnen, 2010)); OECD and World Bank Innovation Indicators; OECD innovation scoreboards ( OECD, 2010; OECD, 2017); and The Global Innovation Index ( Cornell University *et al*., 2017).

Our aim, in this article, is to propose measures that enable hypothesis-driven research on the influence and impact of OS partnerships on a variety of social and economic outcomes, as well as research culture, rigor, diversity, social capital and patient and consumer voice. The set of measures we propose establishes a global basis for collecting and sharing data and will accelerate not only our collective understanding of OS, but provide support and evidence to those contemplating, implementing or monitoring the effects of OS partnerships.

## Methods

We draw on existing methodologies, with the modifications that we discuss below, to develop the set of measures in the proposed measurement toolkit. In particular, we examine the literatures on evaluation of projects, programs, and knowledge transfer. We adopted a three-stage knowledge exchange process to facilitate our development of the toolkit.

The first body of literature assesses whether projects or programs have achieved their anticipated outcomes. This literature relies on logic models to track whether those partnerships deliver outputs that, over the medium and long terms, produce the outcomes promised by those who established the partnership. There are two reasons why logic models are inappropriate for the creation of the measurement toolkit and the set of measures we propose here. First, as noted, logic models are rigid in that they focus on anticipated outcomes within a model rather than exploring foundational questions ( Cooksy *et al*., 2001; Treasury Board of Canada Secretariat, 2012). This narrow focus on anticipated outcomes leaves aside effects that “can be realized by paths other than those presumed by program theory” ( Weiss, 1997). Second, we aim for the toolkit to aid in developing theory rather than applying an established theory. As Weiss notes, “if theory is taken to mean a set of highly general, logically interrelated propositions that claim to explain the phenomenon of interest, theory-based evaluation [i.e., a logic model] is presumptuous in its appropriation of the word.” Weiss writes that logic models derive from an established theory to evaluate whether anticipated outputs actually result from undertaken activities, but not to develop the theory itself ( Weiss, 1997).

Although we do not use formal logic models, we nevertheless acknowledge the importance of developing measures that correspond to potential influences and impact of OS partnerships on R&I systems, diversity, social capital and other critical outcomes. We thus constructed a set of potential hypotheses concerning the influence of OS partnerships, without attempting to eliminate contradictions or alternative pathways. We employed a method of knowledge exchange through which stakeholders come together to identify research questions, jointly construct the measures, collect data and share and analyse that data. In such a method, stakeholders collectively refine knowledge—hypotheses and measures—iteratively until “only the most valid and useful knowledge is left” ( Graham *et al*., 2006). By ensuring a diversity of perspectives in co-creating the set of hypotheses, this process also increases communication and the likelihood of research uptake ( Kothari *et al*., 2011).

We are aware that previously developed measures to describe certain environments have become prescriptive rather than descriptive, often without sufficient analysis of how metrics can establish perverse incentives and perverse side effects ( Cain *et al*., 2005). For example, the use of patent counts and promised licensing revenues from university technology transfer changed from a useful means of comparison to an output measure of performance ( Kim *et al*., 2008). Such practices often lead universities to over-patent and engage in poor licensing practices ( Ryan & Frye, 2017). Using descriptive measures as targets—such as number of patents held—rather than providing a snapshot of current activities, also raises significant ethical concerns over the use and dissemination of measures. These concerns can be partially countered by proposing a large enough set of measures to make it difficult to cherry-pick only a handful of measures that can be gamed. Further, combining quantitative and qualitative measures also reduces the risk of gaming.

We recognize that it is difficult to track causal links between phenomena and ultimate impact ( Council of Canadian Academies, 2013). Beyond the difficulties in establishing causation, OS practice varies based on the setting, problem, available resources and stakeholders. Additionally, internal and environmental features can also lead to multiple pathways and interactions between measures and impacts. Some of these features are difficult to capture, including informal knowledge transfer, relationship building, trust and education of new trainees and expert personnel ( Nicol, 2008). Instead, we expect relationships between OS practices and outcomes to take the form of a contribution chain that acknowledges influence, but shies away from claiming causation.

## A three-stage process

We adopted a three-stage process to implement the knowledge exchange. First, we developed a working definition of OS partnerships based on a review of the literature and of partnerships that consider themselves to be open science. Second, we convened global stakeholders in Washington, DC in October 2017, to map out the ways in which OS partnerships might influence innovation and social and economic outcomes. Third, drawing on these influences and potential outcomes, we brought together experts in measurement, evaluation and empirical studies from a variety of disciplines and countries to develop a prospective set of measures that we propose OS partnerships around the world use to construct data sets.

*Stage 1*

Over the summer of 2017, we conducted an extensive literature review of the academic, policy and grey literature on open science. Based on this, we developed the following definition of open science:

Open science (OS) comprises a set of institutional policies, infrastructure and relationships related to open access publication, open data and scientific resources, and lack of restrictive intellectual and other proprietary rights with the goal of increasing the quality and credibility of scientific outputs, increasing efficiency, and spurring both discovery and innovation. ( Ali-Khan *et al*., 2018a)

*Stage 2*

The global stakeholders we convened in the second stage in Washington, DC in October 2017 included thought-leaders from developed and developing nations, intergovernmental organizations, researchers, governments, science agencies, funders, members from the philanthropic sector, patient organizers, and members from biotechnology, pharmaceutical, and artificial intelligence industries (see extended data, Supplementary File 4 ( Gold, 2019) for a list of participants). After presenting our definition of open science and discussing the example of the MNI, stakeholders together engaged in a series of facilitated discussions asking what success of OS means from the point of view of researchers, governments, industry, philanthropies and patients. The organizers then summarized these discussions and represented them to the group for further discussion and elaboration. Ali-Khan *et al*. (2018a) summarized those discussions, obtained feedback from participants, and published the results. Through these iterative discussions, stakeholders collectively mapped out the different ways that OS partnerships might contribute to innovation and desired or feared social and economic outcomes. Examples of the jointly-created hypotheses included the following: 1) that OS partnerships would simplify and thus increase exchanges of students and postdoctoral fellows between university and industrial labs; 2) that students practicing OS making the transition to tenure track positions would be hindered by not having their own private data set to found their own labs or, alternatively, that these students would benefit by increasing their exposure to a larger network of investigators; and 3) that OS partnerships would increase the quality of data by encouraging researchers to place more emphasis on data quality and reproducibility prior to public exposure or, alternatively, would decrease the quality of data due to the desire and facility of quickly publishing their work and establishing priority.

As these examples illustrate, stakeholders understood the relationship between OS, research, innovation, communities and the public to be complex, and explored different, sometimes contradictory, hypotheses in order to generate, in the third stage, a set of prospective measures that would allow researchers and stakeholders to investigate that relationship. We published the results of that meeting and proposed seven overarching themes for further exploration as follows: 1) Increased quality and efficiency of scientific outputs; 2) Accelerated innovation and impact; 3) Increased trust and accountability of the research enterprise; 4) Increased equity in research; 5) Better opportunities and recognition of early career researchers and youth; 6) Positive economic impact; and 7) Implementation success ( Ali-Khan *et al*., 2018b).

*Stage 3*

At the third stage, we assembled a group of global experts across diverse fields—including innovation measurement and policy, law, public engagement, bibliometrics, economics, business and sociology—in London, UK in May-June 2018 to develop a set of measures to underpin the development of the prospective measurement toolkit (see extended data, Supplementary File 5 ( Gold, 2019) for a list of participants). To provide continuity, we included some participants from the Washington Forum in this workshop. Most participants, however, were new to include individuals with different expertise as well as those involved in other major OS measurement and standard-setting initiatives. The latter included individuals who had worked on the European Commission (EC) OS Monitor, the RAND SGC analysis ( Jones *et al*., 2014), the EC Expert Groups on Indicators and FAIR Data, the TOP Guidelines and the Metric Tide ( Wilsdon *et al*., 2015). We included these individuals to promote alignment and complementary processes between our proposed measures and measurement toolkit with other global OS measurement initiatives.

The goal of this third-stage workshop was to generate prospective measures based on the seven themes produced at the first meeting ( Graham *et al*., 2006). Matching the hypotheses generated in the first workshop to measures enables the testing of hypotheses about the influence of OS partnerships ( Canadian Academies of Health Sciences, 2009; Tracz & Lawrence, 2016). Accordingly, we organized participants into groups corresponding to the seven themes identified in the first workshop. These groups developed working documents with a mixture of quantitative (e.g., counts, revenues, patents, students, survey results, etc.) and qualitative (principally semi-structured interview guides) to provide a nuanced set of data through which to study OS partnerships (see extended data, Supplementary File 6 ( Gold, 2019)).

Following the third-stage workshop, we reviewed and organized the proposed measures. We eliminated duplicate measures and put aside for future work those that were missing critical information (e.g., lack of data source, coding frame, or clear connection to a hypothesis). We sorted (and in some cases adapted to fit a partnership context rather than a country or region) those measures that could be implemented in the study of individual OS partnerships from those that related to general environmental conditions, such as overall government funding or education levels generally. We also recorded measures proposed at the workshops that were specific to countries, specific databases (e.g., databases of academic articles such as PubMed or Web of Science), or that would require the state to compel information disclosure (e.g., by governmental statistical agencies). Finally, we pre-published the measured on the Gates Open Research platform as a document ( Gold *et al*., 2018) and solicited comments for several months from the general community on them. We revised the measures in light of those comments.

We leave these to others to expand and potentially implement in other contexts. We present our outcomes below.

## Results

The outcome is a set of measures that can be collected about OS and non-OS partnerships, and potentially individual institutions or projects, which agree to do so, and the resulting data shared openly. This data will not only create a baseline for analysis but will provide insight into the evolution of research and innovation practices. We divided the measures into separate instruments based on the nature of the measures (quantitative or qualitative), source of the data (participants in the partnership, social science group observing the partnership, or other entity). The seven themes we identified crossed these categories, making them less relevant as an organizing framework of these instruments; nevertheless, we preserved the underlying hypotheses, themes and working group information as metadata to document their origin (see extended data, Supplementary File 3 ( Gold, 2019)).

The measures include the following components:

Toolkit A: A form of annual report of quantitative data related to the partnership, such as publications and data sets (including their persistent unique identifiers such as DOIs), number of students, student employment post-graduation, authorship, investments, etc.;

Toolkit B: A series of semi-structured interview guides to better understand norms, attitudes and understanding across the spectrum of stakeholders involved in the partnership (e.g., do you feel that you derive benefit from your participation in the OS collaboration? What challenges and opportunities does OS present for your business?);

Toolkit C: A form of survey to identify implementation of OS practices within the partnership; and

Toolkit D: A select number of other quantitative measures that require expertise in advanced social-science methods that cannot reasonably be included as part of the annual report in Toolkit A. These include, for example, measures that require linking publications with citations in the academic, grey or patent literatures. We expect teams external to the collaboration (or a distinct unit of the collaboration) to collect these data and share them.

Beyond this set, we identified a non-exhaustive set of measures that can be best implemented by governments, intergovernmental organizations, research funders, agencies, or database owners that are not specific to any one OS partnership (see extended data, Supplementary File 1 ( Gold, 2019)). Finally, we recorded incomplete and rejected measures so that the community may draw on these in the future (see Supplementary File 2 ( Gold, 2019)).

The measures we propose are in plain language and are user-friendly in conformity with best knowledge dissemination practice, thus encouraging user uptake ( Kothari *et al*., 2011). We include definitions, data sources and coding rules, in addition to tracing how we developed the measure and underlying hypotheses that lead to it.

In accordance with good practice, the measures we propose are aimed to be transparent and clear in their coding. We also aimed for the necessary data to be cost effective and easy to collect across a spectrum of OS partnerships. As noted in the methodology section, we combined qualitative assessments to support quantitative evaluations. By publishing these measures, definitions and instruments on an open platform that allows comment, transparent updating and review, we have created the opportunity to continuously update the measures, introduce new ones and retire those that prove difficult to collect or share in practice ( Wilsdon *et al*., 2015).

## Discussion

We developed the set of measures proposed in this article as a necessary step towards the construction of a global measurement toolkit on OS partnerships, which we see as key to understanding changing research and innovation environments and to the role and impact of OS in particular. We anticipate that partnerships around the world will collect and share data on OS practice and outcomes by drawing on our measures. The resulting measurement toolkit will provide researchers with the ability to validate data and improve the measurement toolkit, and to test hypotheses to develop a grounded theoretical understanding of the contributions, positive and negative, of OS partnerships on research, innovation and social and economic life. Stakeholders can also draw on the data to better appreciate their own organizations and operations. Decision-makers in government, industry, universities and community groups will be able to draw on this learning to structure future OS partnerships and to eventually develop logic models through which to assess particular partnerships.

The economic and social influence of OS partnerships may take years to materialize and may be subject to a plethora of diverse influences. While we recognize that OS successes do not happen in a vacuum, careful empirical analysis of OS will nevertheless help researchers identify key determinants of values and benefits of OS. This will allow the community to propose mechanisms to enable OS practice and to define the contribution chain between OS activity and outcomes.

We acknowledge certain limitations to the measures we propose and call on other researchers to investigate and propose improvements. First, while our stakeholders included individuals and institutions from developing countries, data for some of the measures will be easier to collect and most relevant to partnerships in industrialized countries. This is because data sources will likely be more available in industrialized countries and sharing mechanisms, motivations, and barriers to implementation may differ across countries. Specifically, we recognize that data collection in lower-income countries is constrained by lack of resources, weaknesses in institutional organization, and inability of governments and organizations to collect reliable and appropriate data ( Elahi, 2008). Further research is needed to determine the suitability of our proposed measures, to propose additional measures and to investigate ways to access data sources. Second, we derived the indicators predominantly (but not exclusively) from experience with the life sciences, with a particular focus on biomedical science. Whether these indicators are as suitable to other fields such as nanotechnology, information technology, health system analysis, environmental sustainability, arts (digital, visual or performance), agriculture, or history, for example, needs to be investigated.

Finally, to mitigate the dangers of misuse of the measures and their associated data, we encourage those who are using the measures to use them openly and transparently. By doing so, the community can better monitor use of the measures and quickly respond with any concerns arising from their use. 

## Conclusion

Measuring the influence of OS partnerships is important to improving R&I systems because deeper understanding of OS influence will reduce uncertainty about the relative benefits, positive impacts, and negative impacts of OS partnerships. This uncertainty manifests itself in several ways: in a lack of trust in open and public scientific knowledge generation, in a lack of policy frameworks in some countries and by inertia within public research organizations, and in a failure of researchers, public research organizations, communities, or firms to experiment with OS partnerships.

Implementing the set of proposed measures will lead to a data resource to aid in understanding the role of OS partnerships in R&I systems. This data resource might encourage the establishment of OS partnerships by mitigating the uncertainty surrounding OS partnerships, contributing to a better theoretical understanding of OS, and encouraging a shift towards more openness and inclusivity in science. To fully realize this understanding, diverse communities will need to investigate the benefits and drawbacks of using OS approaches using such evidence-based metrics. By doing so, communities can generate an evidence base regarding beneficial impacts and drawbacks of OS, and share data openly as research data. The data therefore should be FAIR (findable, accessible, interoperable and reusable), and “as open as possible but as closed as necessary” ( European Commission, 2016). In order to build a comprehensive data set, it would be advantageous for OS partners to share annual reports and conduct semi-structured interviews and administer the proposed survey at least once every two years. Ideally, we envision that stakeholders will develop an OS partnership that will act as a repository for the data, curate that data, share it and revisit and update, periodically, the measures we propose here. Both the SGC and the MNI have agreed to do so; we invite and welcome other stakeholders to share their data sets should they be willing.

## Measurement Toolkit

### Foreword

This document sets out the measurement toolkit developed in *An Open Toolkit for Tracking Open Science Partnership Implementation and Impact* in order to build a data resource through which to study and, with that knowledge, build assessment tools for open science collaborations. We recommend that partnerships complete and share the results of the Annual Report (Part A) on a periodic basis, which we suggest being once per year. A group independent from the collaboration’s management – to ensure confidentiality of results – ought to administer the semi-structured interviews (Part B) to a representative sample of stakeholders each period. We suggest that the collaboration ought to administer the survey (Part C) at the beginning of the collaboration and periodically thereafter. Finally, we suggest either the collaboration’s administration or an independent group ought to develop the measures in Part D during the same period as for the annual report and after having been given access to the results of the annual report.

We envision that this toolkit be implemented through information technology, rather than through manual data entry, with standard nomenclature (e.g., as to departments and institution names). Two OS organizations, the Structural Genomics Consortium and the Montreal Neurological Institute have agreed to draw upon the toolkit to collect and share data.

## Toolkit A: Open Science Collaboration Annual Report

### Section One: Identity of Partners

1. List the principal academic, community, industrial and governmental partners of the collaboration for the reporting period. For each partner, provide the following details:

1.1. The organizational identifiers;
  1.2. The sector (academic, government, industrial, philanthropic, community, etc.);
  1.3. Whether the organization has an explicit open science mandate and, if so, the scope and nature of that mandate.

### Section Two: Project Outputs

2. List all projects falling within the collaboration for the reporting period. For each project, specify the following:

2.1. Whether the project is new, ongoing or closed;
  2.2. Whether a project plan exists. If a project plan does exist, include the project plan as an appendix to the annual report or, if it is public, provide its persistent identifier (e.g. DOI, registered reports on cos.io/rr/);
  2.3. Whether the project was born open, became open during the project’s process, became open upon the project’s completion, became open after embargo, switched from open to close, or was never open (with open being understood as available to all who desire access with minimal restrictions, e.g. clickwrap agreement); and

3. For each project listed in (2), indicate whether the project includes each of the following:

3.1. Open governance that is available through online strategic and organizational meetings, open minutes, and transparent governance rules;
  3.2. Design processes to create, revise, and comment on projects that are openly available;
  3.3. Project proposals that are openly available;
  3.4. Project and collaboration budgets that are openly available;
  3.5. Output management plans that are openly available;
  3.6. Materials generated through the project that are openly shared to all that ask, except where there is a limited supply of physical materials;
  3.7. Outputs generated by the project that are openly available without further restriction on use, except in well-defined and publicly justified cases, e.g. to protect the privacy of patient or donor information, or the precise location of nesting sites of rare species;
  3.8. Open infrastructure through which one can access and comment on outputs, etc.
  3.9. Review of projects that is openly available;
  3.10. Clear, open and transparent research processes and protocols, such as open lab books, open research meetings, etc. that are openly available;
  3.11. Preregistration of data collection initiatives that is openly available;
  3.12. Ethics reviews and reasoning that are openly available; and
  3.13. (For closed projects or closed aspects of open projects) Provides rationale for why they are closed using a controlled vocabulary in addition to or instead of details.

4. List all publications, including preprints and outreach materials, arising out of the collaboration during the reporting period. For each publication, provide the following details:

4.1. Persistent identifier if available, such as DOI;
  4.2. Full citation including authors, title, journal, source, etc.;
  4.3. Accessibility;
  4.4. Availability in different languages;
  4.5. Whether the journal in which the article is published conforms to TOP guidelines;
  4.6. From which project this publication results; and
  4.7. The standard for machine readability to which the document conforms (e.g., JATS)

5. List all data sets arising from the collaboration in the reporting period. Provide the following information:

5.1. Persistent identifier if available;
  5.2. Full citation;
  5.3. Accessibility; and
  5.4. The standard for machine readability to which the document conforms (e.g., JATS)

6. List any project in the reporting period from question (2) which did not yet result in a publication or in a published data set listed in questions (4) or (5).

### Section Three: Measure of Scale

7. List all external awards, prizes and grants that recognize or directly support OS that were awarded or granted to researchers in the collaboration during the reporting period. For each of these awards, prizes or grants, provide the following details:

7.1. Persistent identifier if available;
  7.2. Title of award, prize or grant;
  7.3. Nature of award (award, grant, prize, etc.);
  7.4. Name of awardee, with number of years following the awardee’s highest degree from the date of convocation of the degree, and name of degree;
  7.5. Organization providing award, prize or grant;
  7.6. Nature of that organization (government agency, industrial, philanthropic, etc.);
  7.7. Period covered by the award, prize or grant; and
  7.8. Value of the award, prize or grant.

### Section Four: Quality of Outputs

8. List all retractions arising out of the collaboration during the reporting period. For each type of output (publications, data), provide the following:

8.1. The total number of outputs;
  8.2. The total number of retractions; and
  8.3. The summary statistics of the reasons for these retractions, using a controlled vocabulary.

9. List of all corrections arising out of the collaboration during the reporting period. For each type of output (publications, data), provide the following:

9.1. The total number of outputs;
  9.2. The total number of corrections; and
  9.3. The summary statistics of the reasons for these corrections, using a controlled vocabulary.

### Section Five: Diversity and Youth Engagement

10. Calculate the number of projects listed in (2) that have at least one non-academic (i.e., not hired to conduct research at a public research organization) stakeholder. Calculate the percentage of projects that include at least one non-academic stakeholder out of all projects.

11. List all early career researchers (ECRs), i.e. PhDs candidates, postdocs, and individuals who have received a PhD within the past 5 years, including postdoctoral fellows, who worked with the collaboration over the preceding five years. For each, provide the following details:

11.1. Period during which the ECRs worked in the collaboration;
  11.2. For each ECR who has left the collaboration, whether the ECR had ever worked or interned outside an academic environment (e.g., in industry, government or civil society) after leaving the collaboration.

### Section Six: Efficiency of Outputs

12. List all legal instruments (e.g. contracts and memoranda of understanding) entered into or renewed in respect to the collaboration during the reporting period. For each contract, provide the following information:

12.1. Persistent identifier if available;
  12.2. Type of instrument (Material Transfer Agreements, research, sponsorship, etc.);
  12.3. Whether the instrument is new or is a renewal;
  12.4. Number of days from the time that the initial instrument negotiations began (ex: request for contract initiated or request to renew) to the execution of the instrument;
  12.5. For each Material Transfer Agreement, the number of days from initial contact to actual transfer of materials; and
  12.6. Whether and to what extent the instrument is open (no claim to intellectual property rights, levels of commitment to open data and open publication, ability to re-share the materials under the same conditions).

13. For each type of contract, calculate the percentage of those contracts that are open.

14. List all new, ongoing or terminated start-ups and spin-outs arising from the collaboration in the reporting period. For each, provide the following details:

14.1. Name of firm;
  14.2. Location (city) of the firm’s head office and locations (cities) of the firm’s other offices;
  14.3. Status of the firm (new, ongoing, or terminated);
  14.4. Relationship between the firm and the partnership (owned by one or more partners, owned by a researcher within the collaboration, etc.)
  14.5. Number of FTEs employed by the firm at year end;
  14.6. A description of the field of operation of the firm;
  14.7. Whether the firm is for profit, not-for-profit, or charitable.

15. For terminated start-ups or spin-outs listed in (14):

15.1. For each of them, calculate the number of months from incorporation to termination.
  15.2. Calculate the average number of months that the terminated firms survived.

### Section Seven: Extended Reach

16. For each item listed in (4) publications – or (5) data – that resulted in a first citation within the reporting period:

16.1. Calculate the number of months between publication and first citation; and
  16.2. Calculate the average number of months from earliest publication to the first citation for both open access publications and all other publications.

17. List all current financial or in-kind contributions to the collaboration by industry or philanthropy during the reporting period other than those listed in (7) to the OS collaboration. For each, provide the following details:

17.1. Persistent identifier if available;
  17.2. The grantor of the investment and the nature of the grantor (firm, foundation, etc.);
  17.3. The value of the investment, specifying cash and in-kind contributions separately; and
  17.4. The period covered by the investment (start and end date).

18. List all for-profit and non-profit firms or organizations that actively partnered with the collaboration during the reporting period. For each, provide the following:

18.1. Persistent identifier if available;
  18.2. Name of Firm;
  18.3. Whether the firm is for-profit or non-profit;
  18.4. The address of the firm’s head office;
  18.5. If the firm has an office in the region, indicate its address and how many employees are employed locally;
  18.6. If available, the firm’s annual revenues;
  18.7. Field of operation; and
  18.8. The contributions of the firm to the collaboration.

### Section Eight: Open Science Engagement

19. List whether the project that results from collaboration during the reporting period has a policy in respect of the following:

19.1. Sharing the research prioritization process (prioritizing certain research questions or methodologies over others);
  19.2. Sharing proposals;
  19.3. Sharing how funding is allocated;
  19.4. Transparency, openness, or inclusion on governance;
  19.5. Sharing budgets;
  19.6. Transparency, openness, or inclusion on research design;
  19.7. Transparency, openness, or inclusion on execution of the research;
  19.8. TOP Guidelines 2 (Data transparency), 3 (Analytic methods (code) transparency), 4 (Research materials transparency, and 5 (Design and analysis transparency);
  19.9. Open access;
  19.10. Sharing of materials and reagents generated by collaboration, sharing of materials and reagents generated by the collaboration through public repositories;
  19.11. Openness of peer review;
  19.12. Openness of how ethics are applied in research decision-making; and/or
  19.13. Openness of rationale for exceptions to open behaviors.

20. Indicate whether the collaboration has a policy of non-open and non-standard, non-open and standard, open and non-standard, or open and standard licensing.

21. Indicate whether the collaboration:

21.1. Has no data preservation, some preservation or a preservation policy;
  21.2. Dedicates resources for long-term preservation of data; and
  21.3. Has its data stored in certified repositories.

22. Select all that describes the collaboration’s level of participation:

22.1. Closed to observation;
  22.2. Observable by invitation (please note whether the invitations issued were public or private);
  22.3. Observable by anyone;
  22.4. Closed to contribution;
  22.5. Contribution by invitation;
  22.6. Contribution by anyone (please note whether the contributions are or can be anonymous or identified);
  22.7. Allows for passive engagement (e.g., use of materials and data without actively participating in the collaboration); and/or
  22.8. Allows for active engagement (e.g., ability to add data or annotations).

23. Indicate whether the collaboration provides training on OS to the following:

23.1. Undergraduate students
  23.2. Graduate students
  23.3. Postdoctoral fellows
  23.4. Continuing professional development for faculty and staff, e.g. clinicians, full-time researchers, research administrators, librarians, legal counsels;
  23.5. Non-academic researchers, community scientists.

## Toolkit B: Semi-Structured Interview Guides

### Description

This is a semi-structured interview guide that is meant to be administered annually by open science (OS) collaborations. The purpose of the interview guide is to gather substantive qualitative measures of the benefits and costs of OS. The guide is designed to include a wide set of OS stakeholders, including full-time academic staff, early career researchers, individuals from the private sector, research participants, and ethics review board members and/or administrators. The interview results will be used for a variety of purposes including, at an aggregated level, to assess the OS partnership, to study OS partnerships in general, to assess quantitative measures of OS impact and so on.

### General Instructions about Consent and Meeting Research Ethics Requirements

Please ensure that, in addition to obtaining consent for use of the raw data by those administering the survey and sharing anonymized or aggregated data generally, that the raw data can be shared with other groups who are operating under a similar protocol and who have obtained ethics approval, even if these other groups are in a different jurisdiction. Also ensure that the nature of the ethics approval and the process that led to it is as openly documented as possible.

### General Questions for All Stakeholders

1. Definition

1.1. What does open science (OS) mean to you?
  1.2. What is the minimal level of openness that you believe is necessary for OS (e.g., open data, open access publications, avoidance of restrictive intellectual property rights, open grants and reviews, etc.)

2. Transparency of Research Output

2.1. Does the OS partnership provide you with information that is useful to your organization and members in a timely and accessible manner? Please give examples, if any, of successful information sharing.
  2.2. What can the OS partnership do to improve knowledge sharing internally and externally?
  2.3. Is the OS partnership structured so that you and your organization can provide input on your information needs, information research questions, priorities, etc.? If not, why not? If yes, how does the OS partnership achieve this?

3. Public Appreciation and Understanding of Research

3.1. Do the collaboration’s partners have a plan to enable public understanding of the research being conducted and of the results?
  3.2. If so, what do you believe to be the effectiveness of this plan?
  3.3. How would you improve this plan?

4. Institutional Attitude to Transparency

4.1. Please describe your perspective on retractions or scientific publications or data sets. Are retractions a sign that the system is working or not working? Should we aim to eliminate or at least reduce retractions?
  4.2. Has the uptake of OS and greater openness in the research process had an effect on the way you think about or handle research errors or retractions? If so, in what ways?
  4.3. Has OS contributed to greater transparency in the research and innovation process? If so, please describe how so. If not, also please describe why not.
  4.4. Do you have any examples?
  4.5. Do you find any changes in the way your peers view retractions and errors? What do you believe is the influence of OS on these views?
  4.6. Do you believe that research transparency has a positive or negative effect on public trust in the scientific-research endeavor? Please explain.

5. Institutional Support for Staff Engaged in OS

5.1. Do you engage in OS practice?
  5.2. If so, do you feel that your institution encourages and supports your efforts to engage in OS practice? In which ways do you feel supported?
  5.3. What could the institution do better to support your engagement in OS practice? Specifically, does your institution’s tenure and promotion policies encourage this engagement?

6. Validation of Quantitative Measures

The following question would be posed to interviewees after providing the results of the collaboration’s annual quantitative audit.

6.1. Do the annual results of the quantitative data collected by the collaboration (such as institutional H-index, publication counts, number of open datasets, patent counts) accurately reflect the research impact of the collaboration’s work? If so, in which ways? If not, what is missing or inaccurate?

7. Awareness of OS within your Institution.

7.1. Have you heard of OS? If so, what does it mean to you?
  7.2. As far as you know, does your institution practice OS? If so, in which ways?
  7.3. How did you hear about OS at your institution?

8. Awareness of OS beyond the institution

8.1 As far as you know, does your institution engage in outreach about OS to the broader community for example to government, civil society or patient organizations, the general public or industry? Please provide some examples (eg through websites, blogs, invited radio interviews, community townhalls or public research festivals, targeted engagement of MPs etc).
  8.2 In what ways have these activities had an impact on awareness of OS outside the institution?
  8.3 What has been the effect of these activities, if any, on the quality or impact of the research conducted by the collaboration? How so?

### Questions for Early Career Researchers

*(PhDs Candidates, Postdocs, and Individuals Who Have Received a PhD within the Past 5 Years)*

9. Attitudes of Early Career Researchers to OS

9.1. Do you practice OS? If so, in which ways do you practice it (consider open grants, open peer review, open budgets, open access publications, open data sets, open laboratory books, open materials exchange, open reagents, etc.)?
  9.2. If you practice OS, what motivated you to do so? How motivated are you: slightly, moderately, or significantly? What demotivates you from practicing OS?
  9.3. Which factors are most important to you when assessing potential employers? How important is the employer’s adherence to OS principles in assessing these factors?

10. New Pathways for Young Investigators

10.1. Do you feel supported in your career by the institution you work for/are affiliated with? If so, please describe reasons for this. If not, also please describe why not.
  10.2. Has your institution developed novel pathways to help you succeed in an OS environment? If so, in what ways?
  10.3. What additional ways could your institution help you succeed?
  10.4. Has the growing adoption of OS practice had a positive or negative effect on your attitude towards your research? Why?

11. Skill Diversity of ECRs Working in OS

11.1. Over the course of your graduate studies, to what extent did you practice OS in your research? Do you have any examples of your engagement with OS from that period?
  11.2. To what extent do you feel that your experience in practicing OS gave you any of the following skills: increased empathy, more varied data analysis skills, greater understanding of other’s perspectives, greater ability to be a lateral thinker, better data curation skills, more transparent research processes, and collaboration skills? How so? Are there other skills not yet mentioned that you believe practicing OS encouraged? How so?

### Questions for Individuals from the Private Sector

12. Growth of Business Models that Use and Support OS

12.1. Does your business draw on any OS outputs? If so, which ones? Please describe the process by which you accessed these outputs.
  12.2. What proportion of your activities are based on OS? How important are these activities to your firm’s success?
  12.3. What challenges and opportunities does OS present for your business? These may include reliance on open access publications, open data sets, product development, identification of markets, identification of partners, quality control, etc.
  12.4. Please describe your business model in respect of your OS activities.
  12.5. How “open” is your business model? How, if at all, do you protect intellectual property?
  12.6. Have you invested (time/money/in-kind/know-how) in an OS initiative?

### Questions for Research Participants

13. Conditions that Contribute to Trust

OS collaborations are partnerships between different institutions, whether between institutions in the public sector or between institutions in each of the public and private sectors, aiming at sharing knowledge and ideas without restrictive rights.

13.1. Have you heard about OS?
  13.2. What do you know of OS? How would you define OS?
  13.3. How did you hear about this? Do you feel that you are sufficiently informed about OS?
  13.4. How are you involved in OS?
  13.5. Do you feel that you derive benefit from your participation in the OS collaboration? If so, in which ways? For example, these may include gaining greater understanding of your contribution, greater knowledge to guide your own activities, financial or other tangible reward, greater networking opportunities, greater sense of involvement in the research or patient community, etc.

### Questions for Ethics Review Board Members and/or Administrators

14. Ethics Committee Preparedness

14.1. Have you encountered OS in the context of your ethics committee work? If so, how did OS come up?
  14.2. What issues, challenges or opportunities has the ethics committee encountered in handling applications that involve OS?
  14.3. To what degree, if any, has OS had an impact on the way you approach project evaluation? In which ways? Do you see this impact as constructive and beneficial or otherwise? Please explain why.
  14.4. Do you believe that your participation in evaluating research ethics applications arising from OS collaborations has altered the way you evaluate ethical concerns? If so, in which ways?
  14.5. In your view, does the increase in OS practices necessitate any changes in the way you conduct ethics reviews? If so, how?
  14.6. Do members of ethics committees need greater training on OS? If so, on what topics and in which ways?

## Toolkit C: Survey for Measurement of Open Science Engagement

### Description

Open science (OS) collaborations aim to reduce transactions costs, increase sharing, and build better connections with communities. This survey is designed to identify best practices for these collaborations and to assess the ways in which the collaboration is open.

### General Instructions for Selecting Survey Participants

Administer to a representative sample of individuals at stakeholder organizations within the collaboration.

### Beneficial Elements

**Table:** 

1. Do you believe these things are beneficial? Click all that apply.				
		Always	Partly	Never
*Open Research Grant Application*				
1.1.	Open research proposals	☐	☐	☐
1.2.	Open reviews of research proposals	☐	☐	☐
1.3.	Open funding decisions and funding allocations	☐	☐	☐
*Open Methodology*				
1.4.	Open governance of projects through online meetings, open minutes, and transparent governance rules	☐	☐	☐
1.5.	Project and collaboration budgets available online	☐	☐	☐
1.6.	Open design processes to create, revise, and comment on projects	☐	☐	☐
1.7.	Clear, open and transparent research processes, such as open lab books, open research meetings, etc.	☐	☐	☐
1.8.	Preregistration of data collection initiatives	☐	☐	☐
1.9.	Open output management plans	☐	☐	☐
1.10.	Availability and use of open infrastructure through which to access and comment on outputs, etc.	☐	☐	☐
*Open Outcomes*				
1.11.	Materials generated by the collaboration are openly shared to all that ask, except where there is a limited supply of materials	☐	☐	☐
1.12.	Where materials are in limited supply, the existence of a clear set of criteria and open governance structure to decide to whom to send materials	☐	☐	☐
1.13.	Outputs generated by the collaboration are openly available without further restriction on use, except to protect the privacy of patient or donor information	☐	☐	☐
1.14.	Outputs, including materials, are subject to open annotations	☐	☐	☐
1.15.	Publications are open access, with open license, open citations and machine actionable full text	☐	☐	☐
1.16.	The outcomes of the collaboration are not subject to intellectual property rights that restrict free and open use and reuse	☐	☐	☐
1.17.	All tools and software are openly accessible and reusable	☐	☐	☐
1.18.	Reporting standards are openly shared	☐	☐	☐
1.19.	Review of projects and of the collaboration are openly available	☐	☐	☐
1.20.	Ethics reviews and reasoning are openly available	☐	☐	☐
1.21.	Any exceptions to openness are transparently and openly shared	☐	☐	☐

### Your Own Activities

**Table:** 

2. Do you intend to engage in the following activities because they are relevant to you or your role? Click all that apply.				
		Always	Partly	Never
*Open Application*				
2.1.	Open research proposals	☐	☐	☐
2.2.	Open reviews of research proposals	☐	☐	☐
2.3.	Open funding decisions and funding allocation	☐	☐	☐
*Open Methodology*				
2.4.	Open governance of projects through online meetings, open minutes, transparent governance rules	☐	☐	☐
2.5.	Project and collaboration budgets available online	☐	☐	☐
2.6.	Open design processes to create, revise, and comment on projects	☐	☐	☐
2.7.	Clear open, and transparent research processes, such as open lab books, open research meetings, etc.	☐	☐	☐
2.8.	Preregistration of data collection initiatives	☐	☐	☐
2.9.	Open output management plan	☐	☐	☐
2.10.	Availability and use of open infrastructure through which to access and comment on outputs, etc.	☐	☐	☐
*Open Outcomes*				
2.11.	Materials generated by the collaboration are openly shared to all that ask, except where there is a limited supply of materials	☐	☐	☐
2.12.	Where materials are in limited supply, the existence of a clear set of criteria and open governance structure to decide to whom to send materials	☐	☐	☐
2.13.	Outputs generated by the collaboration are openly available without further restriction on use, except to protect the privacy of patient or donor information	☐	☐	☐
2.14.	Outputs, including materials, are subject to open annotations	☐	☐	☐
2.15.	Publications are open access, with open license, open citations and machine actionable full text	☐	☐	☐
2.16.	The outcomes of the collaboration are not subject to intellectual property rights that restrict free and open use and reuse	☐	☐	☐
2.17.	All tools and software are openly accessible and reusable	☐	☐	☐
2.18.	Reporting standards are openly shared	☐	☐	☐
2.19.	Review of projects and of the collaboration are openly available	☐	☐	☐
2.20.	Ethics reviews and reasoning are openly available	☐	☐	☐
2.21.	Any exceptions to openness are transparently and openly shared	☐	☐	☐

### Open Practice

**Table:** 

3. Do you believe that the OS collaboration to which this questionnaire refers carries through on the following elements? Click all that apply.				
		Always	Partly	Never
*Open Application*				
3.1.	Open research proposals	☐	☐	☐
3.2.	Open reviews of research proposals	☐	☐	☐
3.3.	Open funding decisions and funding allocation	☐	☐	☐
*Open Methodology*				
3.4.	Open governance of projects through online meetings, open minutes, and transparent governance rules	☐	☐	☐
3.5.	Project and collaboration budgets available online	☐	☐	☐
3.6.	Open design processes to create, revise, and comment on projects	☐	☐	☐
3.7.	Clear, open and transparent research processes, such as open lab books, open research meetings, etc.	☐	☐	☐
3.8.	Preregistration of data collection initiatives	☐	☐	☐
3.9.	Open output management plan	☐	☐	☐
3.10.	Availability and use of open infrastructure through which to access and comment on outputs, etc.	☐	☐	☐
*Open Outcomes*				
3.11.	Materials generated by the collaboration are openly shared to all that ask except where there is a limited supply of materials	☐	☐	☐
3.12.	Where materials are in limited supply, the existence of a clear set of criteria and open governance structure to decide to whom to send materials	☐	☐	☐
3.13.	Outputs generated by the collaboration are openly available without further restriction on use, except to protect the privacy of patient or donor information	☐	☐	☐
3.14.	Outputs, including materials, are subject to open annotations	☐	☐	☐
3.15.	Publications are open access, with open license, open citations and machine actionable full text	☐	☐	☐
3.16.	The outcomes of the collaboration are not subject to intellectual property rights that restricts free and open use and reuse	☐	☐	☐
3.17.	All tools and software are openly accessible and reusable	☐	☐	☐
3.18.	Reporting standards are openly shared	☐	☐	☐
3.19.	Review of projects and of the Collaboration are openly available	☐	☐	☐
3.20.	Ethics reviews and reasoning are openly available	☐	☐	☐
3.21.	Any exceptions to openness are transparently and openly shared	☐	☐	☐

## Toolkit D: Additional Measures of Open Science

We list here measures that require some analysis, such as identifying the citations (including in patents) to outputs. The list that follows requires, as explained below, expansion.

### Patent Citation

1. Citation intensity: Citation intensity weighted by patent family and normalised by research discipline, or technology sector. Citation intensity means the number of third-party patents citing artifacts (academic publications, other publications, blogs, grant applications, laboratory books, data sets, materials, policies) derived from the OS collaboration. Citation intensity is a granular measure and can be assessed at the individual researcher level, department or institutional levels and at a different time period. The Lens.org provides the In4M tool to calculate this number.
  2. Patent Citations to Literature: The number of open access publications and data sets referenced within patents in the reporting period. This can be calculated as the percentage of all artifacts to date arising from the OS collaboration which are cited in patent literature. An alternative measure is the ratio between the average number of citations in patent documents during the reporting period in patents to the collaboration’s artifacts.

*Note: While only including measures on patents, it would be useful to develop indicators similar to the ones above for policy documents. These measures should draw on and complement project outputs as described in Toolkit A, Section Two: Project Outputs.*

### Community and Diversity

3. Equity of Knowledge Production: The percentage of funds and in-kind support made available within the OS collaboration to researchers, firms or communities in non-high-income countries with respect to overall funds. An alternative measure could include comparing how OS and non-OS projects involve marginalized groups within the research process.
  4. Community Engagement: Analysis of project documentations to track the collaboration's community engagement and extent of communication and benefit-sharing with communities. Code 0 if there is no community engagement plan; code 1 if the project plan describes a community engagement plan; and code 2 if the project reports indicate the plan is being followed.

*Note: These measures complement measures described in Toolkit A, Section Three: Measure of Scale and Section Five: Diversity and Youth Engagement, as well as Questions 2, 3 and 13 in Toolkit B.*

## Data availability

### Underlying data

All data underlying the results are available as part of the article and no additional source data are required.

### Extended data

Open Science Framework: An Open Toolkit for Tracking Open Science Partnership Implementation and Impact. https://doi.org/10.17605/OSF.IO/WMPQB ( Gold, 2019).

This project contains the following extended data:

- Supplementary File 1 (Open Science Measures to be Considered by Others)
- Supplementary File 2 (Incomplete and Rejected Open Science Measures)
- Supplementary File 3 (Origin of the Measurement Toolkit)
- Supplementary File 4 (Washington Leadership Forum Participant List)
- Supplementary File 5 (London Workshop Participant List)
- Supplementary File 6 (Notetaking at the London Workshop)

Extended data are available under the terms of the Creative Commons Zero “No rights reserved” data waiver (CC0 1.0 Public domain dedication).
