# Supplementary material for: An open toolkit for tracking open science partnership implementation and impact
Source: Gates Open Res. 2019 Dec 4;3:1442. Originally published 2019 Apr 30. [Version 2] doi: 10.12688/gatesopenres.12958.2 (PMC6904887; doi:10.12688/gatesopenres.12958.2)
Supplement: Peer review history

# Peer review: Reviewer response for version 2

**DOI:** 10.21956/gatesopenres.14243.r28340

**Published:** 2019-12-09

**Authors:** Paolo Manghi

**Affiliations:** 1 Institute of Information Science and Technologies of the National Research Council of Italy (ISTI-CNR), Pisa, Italy

I have read the responses and the revised paper as amended by the authors. I am very happy with the final results and thank the authors for their work.

I confirm that I have read this submission and believe that I have an appropriate level of expertise to confirm that it is of an acceptable scientific standard.

# Peer review: Reviewer response for version 1

**DOI:** 10.21956/gatesopenres.14064.r27761

**Published:** 2019-10-07

**Authors:** Paolo Manghi

**Affiliations:** 1 Institute of Information Science and Technologies of the National Research Council of Italy (ISTI-CNR), Pisa, Italy

**Paper summary:**

 The authors claim that Open Science practices have been introduced as a way to address concerns of trust in research. However OS implementation lacks of standards or even best practices with the result that practitioners apply their own definition of OS. As a step forward, the authors present a toolkit to structurally collect and track over time information about OS partnerships, with the aim of assessing and studying the impact of such partnerships in research, society and innovation. The toolkit is the result of a three-phase process involving experts in the domain and collects data about: (i) quantitative measures collected by OS partnership admins every year; (ii) semistructured set of interviews for stakeholders; (iii) survey from participants in the partnerships; and (iv) quantitative measures for Open Science. The set of measures proposed by the toolkit is the result of merging different understandings of OS, allowing to track and measure participation to particular practices rather than defining a one-size-fits-all interpretation. 

 The exercise focuses on OS partnerships, intended as collaborations where participants all adhere to OS practices. Contracts, policies and infrastructure must therefore find a “way through” to make science reproducible, increase efficiency, and foster innovation. Such virtuous exemplars are invited to use the toolkit in order to track quantitative and qualitative evidence of their OS practices, to provide evidence of the advantages of OS to those organizations, policy-makers, funders, firms which are today skeptical about its cost and benefits trade-off.

 Quite importantly, the acquisition of such data will allow funders, policy-makers, and researchers to define appropriate and better indicators of success in terms of OS practices and inherent scientific, social, economic impact.

**Feedbacks:**

*Definition of Open Science*: The definition adopted in this paper focuses on open access and sharing of outcomes such as literature and data. According to other interpretations, Open Science includes software as a distinct first-class citizen, beyond literature and data. Recent developments are showing also how "thematic services/infrastructures" (aka digital laboratories) are key for reproducibility and should be part of the "research outcome package". Semantic links between scientific products (article-data, article-software) are also extremely important and not always provided; the absence or obsolescence of such links compromises reproducibility and discoverability of science. Moreover, I also came to appreciate definitions of Open Science that include the notion of "open collaboration", which implies again a discussion on sharing and access rights, but imply the adoption of methodologies and tools (e.g. Virtual Research Environments) opening to collaboration during the research life-cycle, so while experiments are still being performed, and not just before or afterwards. 

 These observations do not represent reservations about the article, but of course impacts in the kind of measures that are proposed and could/should be included in the toolkit. I see the toolkit as a very good milestone in the Open Science roadmap, I just fear it could be a missed opportunity not to include some key aspects in the current measures.

*About measures*

- It is not very clear why the measures in Toolkit D about patents do not find a sub-section in Toolkit A about patent measures.
- For the aforementioned reasons, I would have appreciated a section on open source and software publishing practices and relative measures.
- If Toolkit D is intended as list of functions to be used to calculate indicators, then I would suggest to define an explicit link between indicators in Toolkit D and the measures in all toolkits A, B, C. For example, social and economic indicators are key to "depict" the measures in Toolkit B, otherwise only expressed in heterogenous narrative forms (still very important, but hard to process, evaluate and confront). Ideally, around the toolkits, scientists should fire an iterative process of analysis of responses and identification of indicators to be added to Toolkit D.
- The fact the proposed measures were defined in the context of life-science is specified as an aside note in the Discussion section. I would say this is a quite key piece of information, that should emerge sooner in the text (if not in the title/sub-title). It is also very important to acknowledge the fact Open Science cannot have ONE definition or ONE interpretation as different disciplines have different research life-cycles and practices (the discussion is very similar to the one undertaken under the FAIR initiatives). In fact, the Toolkits may well introduce the notion of "Community profile", intended as the specific set of measures that are of interest to given communities, to be identified and fine-tuned over time by means of the Toolkits. Such community perspective could be a good incentive for using the Toolkits which could become the means where policies, standards, and best practices can be collaboratively defined by research communities.

*Others*

- To foster Open Science it is also important to provide evidence of the fact its implementation takes to better, or at least equivalent, results when compared to non-Open Science collaborations, especially from the researcher’s point of view. Are we aware of any similar toolkits, adopted in more traditional scientific settings, whose data can be used to compare the outcomes of OS and non-OS partnerships?
- The impact of Open Access and Open Science mandates on OS partnerships is also another important evidence that could be collected that does not seem to be directly addressed by the Toolkit A and B.

I confirm that I have read this submission and believe that I have an appropriate level of expertise to confirm that it is of an acceptable scientific standard, however I have significant reservations, as outlined above.

# Response

**Published:** 2019-10-26

**Authors:** Richard Gold

**Affiliations:** McGill University, Canada

Thank you very much for this excellent review and suggestions. On behalf of my co-authors, I am pleased that you find the article a useful step in the progress of understanding the effects of open science. As you note, we see our proposed toolkit as a first step that will need testing and elucidation over time. From my perspective, the toolkit represents a snapshot of thinking on open science that represents a consensus among a particular group of actors. We anticipate that others will build on and refine it over time. In the meantime, we seek partnerships to begin collecting data using it. As other researchers add other measures to the toolkit, we anticipate that those partnerships will collect data on those measures as well. It is nevertheless important that collaborations start collecting data immediately so that the entire community has access to the data.

Given this and the nature of the process we used, the article and this version of the toolkit are now fixed artifacts. We encourage other researchers to build on them and add additional measures and critique the measures we proposed. Thus, I will respond to your very helpful comments but will not alter the toolkit itself.

Here are my specific responses to your comments.

*About measures:*

- **Comment:** It is not very clear why the measures in Toolkit D about patents do not find a sub-section in Toolkit A about patent measures. **Response:** We could not reach consensus on the sources of data to use nor whether the measures ought to be restricted to patents. We encourage others to further develop these measures so that they can be added at a later time. We agree that, once refined, they could add to Toolkit A.

 

- **Comment:** For the aforementioned reasons, I would have appreciated a section on open source and software publishing practices and relative measures. **Response:** The article was focused on open science partnerships rather than publishing practices overall. I believe that the comments in your review complement the article well and encourage readers of the article to also read and consider your comments.

 

- **Comment:** If Toolkit D is intended as list of functions to be used to calculate indicators, then I would suggest to define an explicit link between indicators in Toolkit D and the measures in all toolkits A, B, C. For example, social and economic indicators are key to "depict" the measures in Toolkit B, otherwise only expressed in heterogenous narrative forms (still very important, but hard to process, evaluate and confront). Ideally, around the toolkits, scientists should fire an iterative process of analysis of responses and identification of indicators to be added to Toolkit D. **Response: **As noted above, we were not able to reach consensus on the measures in Toolkit D. As they all need more work and could significantly alter in the process, I suggest it is premature to link them up at this time to Toolkits A, B and C.

 

- **Comment:** The fact the proposed measures were defined in the context of life-science is specified as an aside note in the Discussion section. I would say this is a quite key piece of information, that should emerge sooner in the text (if not in the title/sub-title). It is also very important to acknowledge the fact Open Science cannot have ONE definition or ONE interpretation as different disciplines have different research life-cycles and practices (the discussion is very similar to the one undertaken under the FAIR initiatives). In fact, the Toolkits may well introduce the notion of "Community profile", intended as the specific set of measures that are of interest to given communities, to be identified and fine-tuned over time by means of the Toolkits. Such community perspective could be a good incentive for using the Toolkits which could become the means where policies, standards, and best practices can be collaboratively defined by research communities. **Response:** Perhaps we ought to have highlighted our focus on life sciences earlier, but our intention is to create a more general toolkit that can be used to compare effects across fields. We anticipate that different fields and communities will draw on the toolkit to develop community specific measures. However, we urge all communities to collect data on as many of the measures as possible so that there exists a set of measures that allow for comparison.

*Others:*

- **Comment:** To foster Open Science it is also important to provide evidence of the fact its implementation takes to better, or at least equivalent, results when compared to non-Open Science collaborations, especially from the researcher’s point of view. Are we aware of any similar toolkits, adopted in more traditional scientific settings, whose data can be used to compare the outcomes of OS and non-OS partnerships? **Response:** We conducted an extensive literature search prior to preparing the article, drawing on the expertise of all those who attended the workshops. We identified some measures, as noted in the article, that we brought into the toolkit but none of the existing sets of measures was sufficient for the purposes in their existing forms.

 

- **Comment:** The impact of Open Access and Open Science mandates on OS partnerships is also another important evidence that could be collected that does not seem to be directly addressed by the Toolkit A and B. **Response:** We looked at actual practices within the partnerships rather than the reasons that motivated adoption of those practices. We sought to encourage the collection of data on those actual practices. To the extent that a mandate was adopted formally or informally within the partnership, they are captured by the detailed questions in Toolkit C.

# Peer review: Reviewer response for version 1

**DOI:** 10.21956/gatesopenres.14064.r27758

**Published:** 2019-09-04

**Authors:** Jon Tennant

**Affiliations:** 1 Center for Research and Interdisciplinarity, Paris, France; 2 Institute for Globally Distributed Open Research and Education (IGDORE), Paris, France

The authors present a new toolkit and dataset for use within the growing open science landscape, centred around tracking the impact of open science collaborations. As such, it represents a timely article, especially during a time when there is so much complexity and innovation to follow. Given the immense collective expertise of the authors, I’m not sure what more my review here can add, but let’s see anyway!

 Note, as a conflict of interest I personally know several of the authors of this study. Also, I generally support ‘open science’, and therefore it is of interest to me that papers that help to advance the cause or generate further discussion get published. Most of my comments here are simply questions that I hope the authors consider in order to improve the argumentation style within the paper.

 Abstract:

- The abstract is clear, concise, and conveys the key points of the manuscript.

Introduction:

- Who is ‘we’ in the first sentence? I can think of a few people who might object to this. There also feels like a gap between the first and second sentences. What does research have to do with the first sentence?
- Wasn’t there a recent report showing that public trust in research is actually on the rise? I believe that one of the authors (BN) tweeted about it, and its link to open science.
- Is it *just *the incentive system that leads to QRPs and irreproducibility?
- Are governments really adopting OS practices? Or just supporting them?
- Is OS just a set of practices? Or something more fundamental too, like a set of principles and values?
- What does ‘restrictive’ IP mean?
- I feel when describing what OS practices are, more people than just the lead author of this paper could perhaps be cited? More people have been working on this since before 2016.
- Is OS being non-standardised necessarily a bad thing? What about its consideration as a ‘boundary object’? And what about something like the FOSTER taxonomy (Moore, 2017 ^1^)?
- Are there any other ‘open science toolkits’ that exist already? Or anything similar?

OS partnerships:

- Are there any examples from outside of Canada you can think of?
- Is there really such little evidence surrounding the benefits of OS?
- Were any librarians included as stakeholders?

Literature review:

- It feels strange not to cite McKiernan *et al.* (2016 ^2^) here as evidence of the positive effects of OS.
- Also what about things like the OA citation advantage?

Methods:

- How were the literature selected for assessment?
- I feel the bit on logic models is a bit of a tangent, especially as in the next paragraph you mention that you don’t actually use them.
- How were stakeholder groups selected? Can you give any details on their demographic representation, or speciality etc? For example, I note in your author list that virtually all authors are from North America or western Europe, with just one from Africa and one from South America. None are from Asia. I feel that you need to be very careful in claiming representativity in this article, and also discuss the effects that your selection of expertise here might have in limiting the epistemic diversity and outcomes of this project. As a suggestion, I would suggest even having this paper/whole project critically examined by more experts from the developing world (who definitely exist), possibly as a toolkit 2.0, so that a more diverse demographic is more fairly represented. I feel this is important if we want to move away from (accidentally) reinforcing western hegemony through open science. I note that the participant list is a bit different from the final author list.

A three-stage process:

- Is this part of the Methods section? Because the heading styles match at the moment.
- Was the literature review systematic? How were the literature selected? Do you have a list?
- What does ‘restrictive’ mean here in your definition?
- How does your definition differ from other definitions of OS?
- For the seven themes, these are all positive too. Is it unanimous that OS definitely only has overall positive benefits? There aren’t any potential drawbacks at all?
- For your global experts, same issues as above.
- Which participants from DC attended the London workshop? It seems again that participation is *very* focused on European and North American-led initiatives. I know from personal experiences in this space that this is not necessarily the fault of the project leads here. However, I think you definitely need to be clear about who was *not* in the room too. And whether there are any similar non-western projects or anything that this could build on. Alternatively, if there is nothing in the ‘developing world’ that is similar, perhaps this is even worth commenting on and highlighting as something to be actively aware of in the future.
- Was there any discussion too about potential sources of bias in the datasets? For example, Web of Science is mentioned, but known to be heavily biased against research from the developing world again. If this toolkit is meant to be a global one, then I feel some mention of this could be needed.

Results:

- Generally, I think the 4 different toolkits are very well thought out and designed.
- Is the toolkit only available in English? I think you know where I’m going with this.

Discussion:

- I’m glad to see that the authors want to see the toolkit revised. I think this is important, especially given the dynamic nature of open science at the present (and presumably the future).
- The mention here that this is geared towards the life sciences needs to be made much clearer and earlier on, I feel. It popped out as a surprise here!

Conclusion:

- Do you think that maybe government funders, publishers, researchers and librarians should maybe all come together to build something like a shared open data infrastructure for open science? Not one that is closed, proprietary, and based on heavily biased data sources like some other services. I think this would be vastly superior and key, as such data could have potentially great uses beyond simply evaluation.

Other general thoughts:

- Measure all the things! Reading through this, and looking at the four main elements, pretty much all of this is about data gathering and surveying open science collaborations. I wonder, do the authors feel this might have any unintended consequences? Administrators love metrics, and I wonder if for example, such data could be used in potentially deleterious ways to impact researchers, their work, or careers. Alternatively do you feel that if Goodhart’s Law comes into practice, such that researchers start ‘gaming’ metrics in a way that is beneficial to themselves, but also (open) science, do you think this is a good thing? I know this is mentioned in the methods section briefly, but dismissed a little too easily. I think it would be a good idea to be more critically reflective on such potential issues.
- Some of the keywords are in the title, and so probably redundant for SEO things.
- For the toolkit, do you see there being any associated costs with managing it?
- Who is actually supposed to gather the data for the toolkit? I can imagine many researchers would be thrilled to have such an additional administrative burden!

Overall, I think this is a valuable resource to have created, and congratulate the authors on such a large effort. I hope the comments here are useful and help to improve the MS a bit.

I confirm that I have read this submission and believe that I have an appropriate level of expertise to confirm that it is of an acceptable scientific standard.

## References

[1] : A genealogy of open access: negotiations between openness and access to research. *Revue française des sciences de l’information et de la communication*.2017; 10.4000/rfsic.3220 10.4000/rfsic.3220

[2] : How open science helps researchers succeed. *eLife*.2016;5: 10.7554/eLife.16800 10.7554/eLife.16800 PMC497336627387362
